# Effects of the Implementation of Intelligent Technology for Hand Hygiene in Hospitals: Systematic Review and Meta-analysis

**DOI:** 10.2196/37249

**Published:** 2023-05-29

**Authors:** Yi Zhang, Xiangping Chen, Yuewen Lao, Xiaobin Qiu, Kang Liu, Yiyu Zhuang, Xiaoyan Gong, Ping Wang

**Affiliations:** 1 Sir Run Run Shaw Hospital Zhejiang University School of Medicine Zhejiang China

**Keywords:** hand hygiene, intelligent technology, meta-analysis, systematic review, multidrug resistance, infection

## Abstract

**Background:**

The World Health Organization recommends regular hand hygiene monitoring and feedback to improve hand hygiene behaviors and health care–associated infection rates. Intelligent technologies for hand hygiene are increasingly being developed as alternative or supplemental monitoring approaches. However, there is insufficient evidence regarding the effect of this type of intervention, with conflicting results in the literature.

**Objective:**

We conduct a systematic review and meta-analysis to evaluate the effects of using intelligent technology for hand hygiene in hospitals.

**Methods:**

We searched 7 databases from inception to December 31, 2022. Two reviewers independently and blindly selected studies, extracted data, and assessed the risk of bias. A meta-analysis was performed using the RevMan 5.3 and STATA 15.1 software. Sensitivity and subgroup analyses were also conducted. Overall certainty of evidence was appraised using the Grading of Recommendations Assessment, Development, and Evaluation approach. The systematic review protocol was registered.

**Results:**

The 36 studies comprised 2 randomized controlled trials and 34 quasi-experimental studies. The included intelligent technologies involved 5 functions: performance reminders，electronic counting and remote monitoring，data processing，feedback，and education. Compared with usual care, the intelligent technology intervention for hand hygiene improved health care workers’ hand hygiene compliance (risk ratio 1.56, 95% CI 1.47-1.66; *P*<.001), reduced health care–associated infection rates (risk ratio 0.25, 95% CI 0.19-0.33; *P*<.001), and was not associated with multidrug-resistant organism detection rates (risk ratio 0.53, 95% CI 0.27-1.04; *P*=.07). Three covariates, including publication year, study design, and intervention, were not factors of hand hygiene compliance or hospital-acquired infection rates analyzed by meta-regression. Sensitivity analysis showed stable results except for the pooled outcome of multidrug-resistant organism detection rates. The caliber of 3 pieces of evidence suggested a dearth of high-caliber research.

**Conclusions:**

Intelligent technologies for hand hygiene play an integral role in hospital. However, low quality of evidence and important heterogeneity were observed. Larger clinical trials are required to evaluate the impact of intelligent technology on multidrug-resistant organism detection rates and other clinical outcomes.

## Introduction

Hand hygiene (HH) refers to washing hands with soap and water, or other detergents containing an antiseptic agent to reduce or inhibit the growth of microorganisms [1]. The World Health Organization (WHO) regards HH as the most effective way of reducing the transmission of pathogens that cause health care–associated infections (HCAIs) and promotes HH in the Clean Care is Safer Care program [1]. In 2009, the WHO summarized the 5 key moments of HH and recommended 2 standard HH techniques in the guidelines [1]. However, hand hygiene compliance (HHC) and HH quality remain suboptimal, even during the COVID-19 pandemic [2]. The WHO found that the average baseline HHC rate among health care workers (HCWs) was only 38.7% [1]. Szilágyi et al [3] reported that only 72% of HCWs could adequately clean all hand surfaces after HH training. Irregular hand hygiene behavior will significantly increase the risk of HCAIs. The impact of HCAIs involves prolonged hospital stay, long-term disability, increased resistance of microorganisms to antimicrobials, massive additional financial burden, high costs for patients and their families, and excess deaths [4]. The WHO recommends regular HH monitoring and feedback to improve HH behaviors and control HCAIs [5].

Direct observation by trained auditors is regarded as the gold standard for HH monitoring and is still widely used in a variety of health care settings [6-8]. However, the process of direct observation is laborious, time-consuming, and costly and may lead to inaccurate data due to the Hawthorne effect (HHC rates are higher during observation but return to baseline as soon as observation stops) [9-11]. Recent work by Purssell et al [12] attempted to quantify the Hawthorne effect by analyzing 9 studies comparing covert with overt measurement and concluded that covert monitoring may give a better estimate of HHC. Therefore, HH behaviors cannot be improved well because of the inherent limitations and bias of direct observation [13,14].

A new method for more accurately measuring and better improving HH is a necessary step in making significant promotions in hospitals [15]. An increasing number of intelligent technologies for HH have been developed as alternative or supplemental monitoring approaches over the last few years [15,16]. Recent advances in sensor technologies and algorithms have also contributed to the development of new intelligent technologies for HH. The devices and technologies include electronic counters, pressure sensors on alcohol-based hand rub dispensers, doorway entry or exit monitors, infrared beacons, and electronic badges [16-18]. McMullen et al [19] used 3-year electronic monitoring systems in 12 hospitals that found a 23% increase in hand hygiene performance.

Although studies have investigated the effectiveness of different intelligent technologies for hand hygiene in hospitals, to the best of our knowledge, only 2 reviews were attempted to summarize the evidence resulting from these studies [11,20]. However, the findings of intelligent technology effects were inconsistent or even contradictory among different studies, and most previous studies only focused on the impacts of intelligent technology on HHC [21,22]. As such, we reviewed the literature and conducted a systematic review and meta-analysis to ascertain the effects of intelligent technology interventions on clinical and process outcomes.

## Methods

### Registration

We followed the PRISMA (Preferred Reporting Items for Systematic Reviews and Meta-Analyses) guidelines to report our systematic review and meta-analysis [23]. Our PRISMA checklist is provided in Multimedia Appendix 1 [23]. The protocol of our study was registered in the PROSPERO (International Prospective Register of Systematic Reviews).

### Search Strategy

We adhered to the PRISMA-S (PRISMA Search Reporting Extension) checklist [24]. The reviewer (YZ) searched the CENTRAL and CDSR (via the Wiley platform), MEDLINE (via the PubMed platform), CINAHL (via the EBSCO platform), Web of Science Core Collection (via the Web of Science platform), Embase (via the Ovidsp platform), and Chinese Academic Journal (via the CNIK platform) databases from inception to December 31, 2022, with no restrictions on language or year of publication. The search strategy included terms related to hand hygiene and intelligent technology. Our strategy was developed in consultation with a medical research librarian. Textbox S1 in Multimedia Appendix 2 details the search strategies of databases. ClinicalTrials.gov and the World Health Organization International Clinical Trials Registry Platform were searched for ongoing and unpublished trials. In addition, we manually searched the references of the collected articles and systematic reviews.

### Eligibility Criteria

Articles were eligible for inclusion in the meta-analysis if they met all of the following criteria: (1) were randomized controlled trials (RCTs) or quasi-experimental studies; (2) included HCWs or adult patients (18 years or older) as participants; (3) evaluated the effectiveness of an intelligent technology–related intervention alone or in combination with usual care compared with placebo or usual methods; and (4) reported at least one clinical end point such as HHC rates, HCAIs rates, or multidrug-resistant organism (MDRO) detection rates (Table 1).

Articles were excluded if they met any of the following criteria: (1) failed to provide the full text and the abstract provided insufficient information, (2) had insufficient or incorrect data, or (3) were duplicate studies.

**Table 1:** Inclusion and exclusion criteria.

Variable		Inclusion criteria
**Study characteristics**		
	Study design	RCTs^a^ or quasi-experimental studies
	Publication type	Full-text journal publications and unpublished dissertations or theses
	Publication year	No limit
	Language	No limit
**PICO^b^ framework**		
	Population	HCWs^c^ or adult patients (18 years or older) as participants
	Intervention	An intelligent technology–related intervention alone or in combination with usual care
	Comparison	placebo or usual methods
	Outcomes	At least one clinical end point such as HHC^d^ rates, HCAIs^e^ (CLABSIs^f^, VAP^g^, SSIs^h^, and CAUTIs^i^) rates, or multidrug-resistant organism (MRSA^j^, CRE^k^, VRE^l^, CR-AB^m^, MDR-PA^n^, and PDR-PA^o^) detection MRSA rates

^a^RCT: randomized controlled trial.

^b^PICO: population, intervention, control, and outcomes.

^c^HCW: health care worker.

^d^HHC: hand hygiene compliance.

^e^HCAI: health care–associated infection.

^f^CLABSI: central line–associated bloodstream infection.

^g^VAP: ventilator-associated pneumonia.

^h^SSI: surgical site infection.

^i^CAUTI: catheter-associated urinary tract infection.

^j^MRSA: methicillin-resistant *Staphylococcus aureus*.

^k^CRE: carbapenem-resistant *Enterobacter*.

^l^VRE: vancomycin-resistant *Enterococcus*.

^m^CR-AB: carbapenem-resistant *Acinetobacter baumannii*.

^n^MDR-PA: multidrug-resistant *Pseudomonas aeruginosa*.

^o^PDR-PA: pandrug-resistant *Pseudomonas aeruginosa*.

### Study Identification and Data Extraction

The data management software EndNote X9 (Clarivate Analytics) was used. Two reviewers (XBQ and KL) independently screened the titles and abstracts for eligibility. Articles were retrieved in full upon request from the reviewers. Then, the reviewers independently screened the full texts and resolved disagreements through discussion. If they could not reach an agreement, another author (YYZ) was consulted, and a decision was made by a majority vote.

Data were extracted independently by 2 authors (YZ and YWL) using predetermined forms (Table S1 in Multimedia Appendix 2). The following data were collected: authors, year of publication, country, study design, setting, participants, intelligent technology intervention, data collection period, and study outcomes.

### Quality Assessment

Two reviewers conducted the risk of bias assessment using the Cochrane risk-of-bias methodology [25] for RCTs and the ROBINS-I (Risk Of Bias In Non-randomised Studies—of Interventions) tool for nonrandomized intervention studies [26]. The assessment tools were both developed by the Cochrane Collaboration. In addition, we used the Grading of Recommendations, Assessment, Development, and Evaluation (GRADE) approach to classify the certainty of evidence into high, moderate, low, or very low for each outcome [27].

### Data Synthesis and Statistical Analysis

Meta-analysis was performed using Review Manager (RevMan, Version 5.3; the Nordic Cochrane Centre, the Cochrane Collaboration, 2014, Copenhagen, Denmark) and STATA Version 15.1 (version 15.1.629; StataCorp). Heterogeneity among studies was assessed using the chi-square test, and *Ι*^2^ values were used to determine heterogeneity across studies. A random- or fixed-effects model was used to calculate the pooled effect sizes and corresponding 95% CIs based on the heterogeneity. If *Ι*^2^≤50%, which represented homogeneity, fixed-effects models were selected. If *Ι*^2^>50%, which indicated substantial heterogeneity of the effects, random-effects models were applied [28]. For continuous data, the mean difference and 95% CI were assessed for the pooled outcomes, and for dichotomous outcomes, the odds ratio and 95% CI were used in accordance with intent-to-treat principles. A forest plot was generated to represent the meta-analysis results. To gain insight into the sources of substantial heterogeneity, prespecified meta-regression was conducted with the following covariates: article publication year, study design, setting, and intelligent technology intervention (different components of the intelligent technology system). The sensitivity analysis was performed by eliminating studies to assess whether the results were stable [29]. If more than 10 studies were included in the analysis of outcomes, funnel plots were constructed to identify publication bias by Egger tests (with *P*<.05 considered significant) [29].

## Results

### Description of Search and Study Characteristics

**Figure 1:**
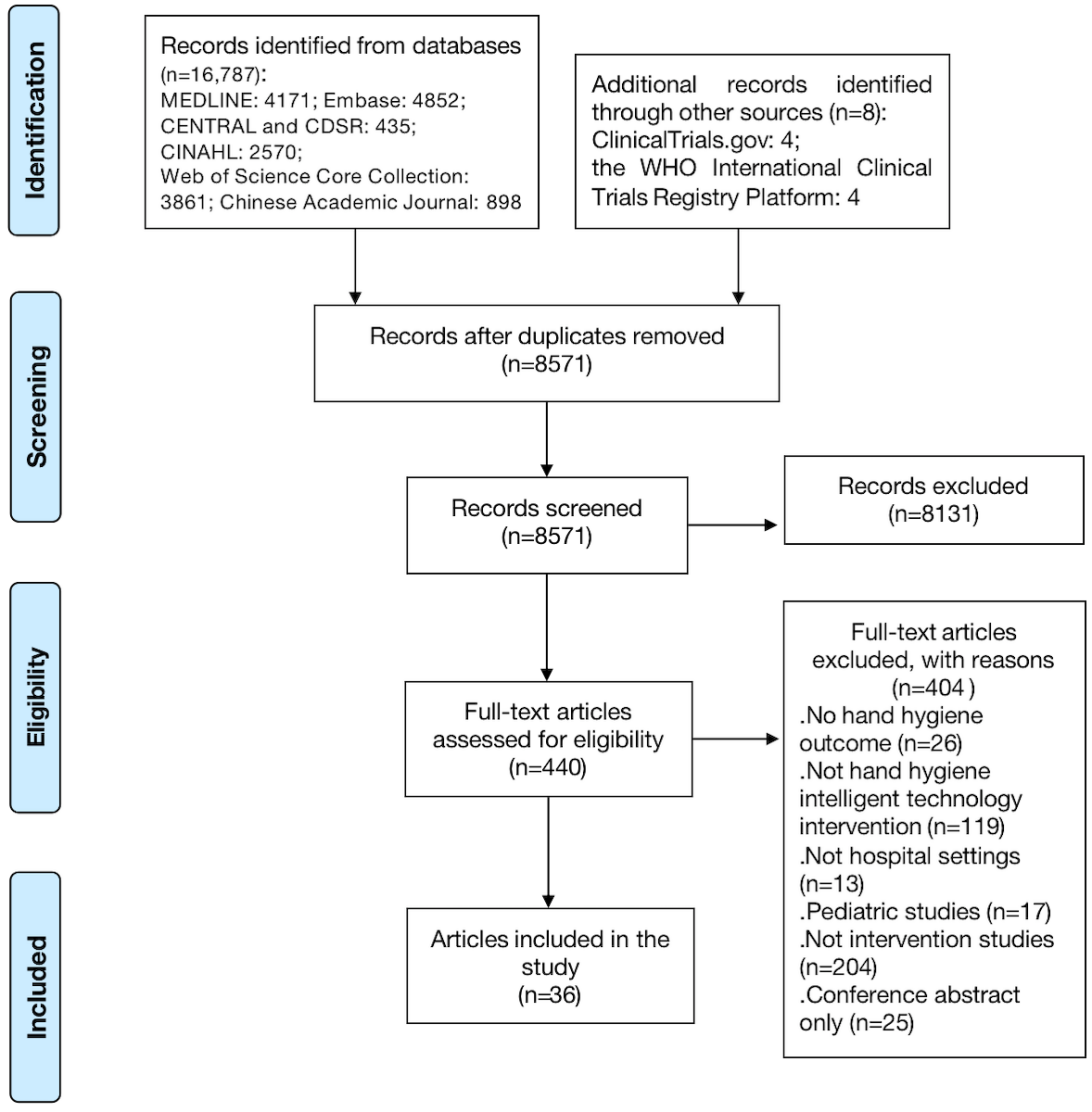
PRISMA (Preferred Reporting Items for Systematic Reviews and Meta-Analyses) flow diagram of study selection. WHO: World Health Organization.

The PRISMA flowchart depicts the extensive search process (Figure 1). We identified 16,791 articles and reviewed 8571 unique titles and abstracts (after removing duplicates across databases) and 440 full-text articles, with 36 studies meeting the predefined eligibility criteria [15,20,22,23,30-61]. Study characteristics related to the population, interventions, and outcomes for the 36 included studies are provided in Table S2 in Multimedia Appendix 2 [15,19,21,22,30-60,62]. The 36 unique articles included 2 (6%) RCTs [53,54] and 34 (94%) quasi-experimental studies [15,19,21,22,30-52,55-60,62] (8 non-RCTs and 26 one-group pretest-posttest quasi-experimental designs). Studies were published between 2013 and 2022, apart from 2 studies published in 2008 [48,58]. Twenty-five (69%) studies were published in the past 5 years. The demographic information of participants was provided in only 5 (14%) studies. Most studies (31/36, 86%) recruited HCWs from hospitals or clinics.

#### Setting

Twenty-one (58%) studies evaluated the impact of intelligent technology in intensive care units, 12 (33%) studies evaluated multiple departments in the hospitals, and only 2 (6%) evaluated operating rooms. The remaining studies involved only different types of departments.

#### Interventions

The included intelligent technology interventions could be grouped into the following five components: (1) performance reminders: HCWs were promoted either through wearable devices, electronic communications, or other methods to remind them about HH; (2) electronic HH counting and remote monitoring: devices were installed on handwashing equipment to remotely monitor and capture HH data; (3) data processing: data were uploaded to a database and analyzed; (4) feedback: compliance feedback was provided to staff via mobile messages, emails, or other methods; and (5) education: an educational program on correct HH procedures was provided. Only 4 (11%) studies reported on single-component interventions, and most of the studies (72%) involved more than 3 component interventions. Components 1, 2, 3, and 4 were widely used in the design of intelligent technologies for HH.

#### Outcomes

Eleven (31%) studies evaluated more than one result of the impact of intelligent technology on HH. HHC was the most assessed outcome in the included studies, with 22 (22/29, 76%) articles assessing HHC when entering and leaving unit areas, 6 (6/29, 21%) articles assessing HHC at the WHO’s 5 moments (WHO moment 1: before touching a patient; WHO moment 2: before clean or aseptic procedures; WHO moment 3: after body fluid exposure risk; WHO moment 4: after touching a patient; WHO moment 5: after touching patient surroundings), and 1 (1/29, 3%) article assessing HHC at WHO moments 1 and 4.

### Risk of Bias and Level of Evidence

Table S3 in Multimedia Appendix 2 [15,19,21,22,30-52,55-60,62] provides a summary of the risk-of-bias assessment for all included nonrandomized intervention studies (n=34) based on the ROBINS-I tool. Nine articles were evaluated as having serious biases, and the other studies were evaluated as having moderate biases. The risk-of-bias assessment for the RCT studies is provided in Figure S1 in Multimedia Appendix 2 [53,54]. Two studies all showed moderate biases [53,54]. Overall, the quality of the studies was deemed as having high bias.

### Pooled Outcomes

#### Hand Hygiene Compliance

**Figure 2:**
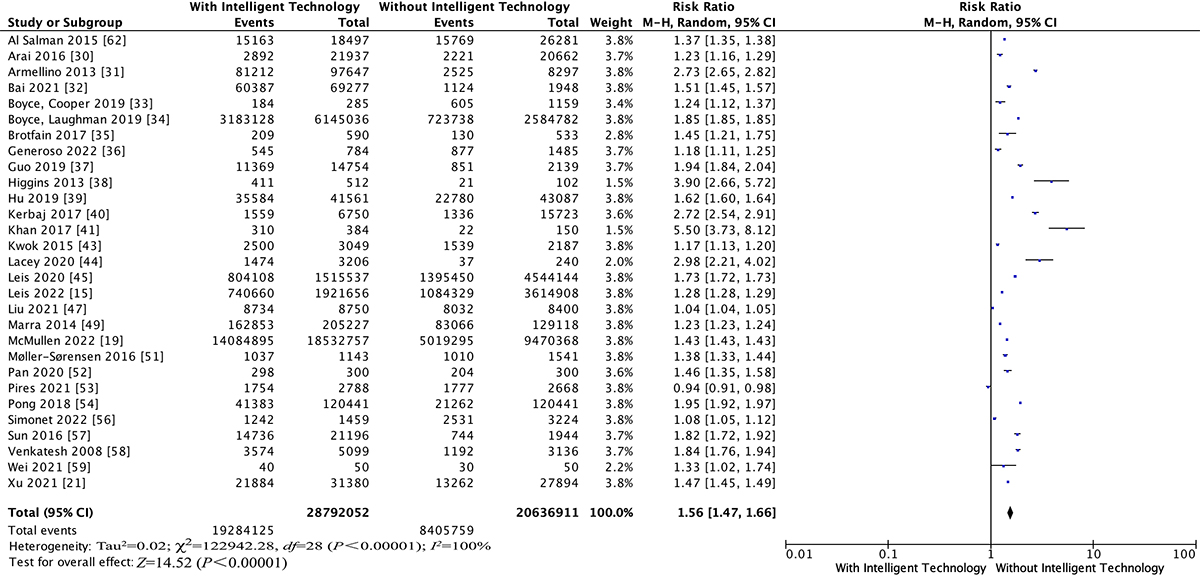
Forest plots for the outcome of hand hygiene compliance.

A total of 29 of the 33 included studies reported on HHC. A random-effects model was performed because of the significant heterogeneity for this outcome (*I^2^*=100%, *P*<.001; Figure 2) [15,19,21,30-41,43-45,47,49,51-54,56-59,62]. The pooled risk ratio (RR) of HHC was 1.56 (95% CI 1.47-1.66, *P*<.001; Figure 2). The results of the meta-regression analysis indicated that the prespecified covariates had no effects on HHC (Table 2). The results of sensitivity analysis obtained by deleting 6 studies with less than 1000 HH during intervention were not significantly different from the combined values of all studies (Table 3). The results of the Egger test showed no publication bias (*t*=−1.23, *P*=.23>.05; Figure S2 in Multimedia Appendix 2). Using the GRADE summary of evidence, the quality of evidence was very low and downgraded for indirectness, high risk of bias, and imprecision.

**Table 2:** Meta-regression analysis of multiple covariates for HHC^a^ and HCAI^b^ rates.

Outcomes and covariate		Regression coefficient	95% CI	*P* value
**HHC**				
	Year	−1.913	−0.6951 to 0.0312	.44
	Setting	0.0730	−0.0103 to 0.1564	.08
	Design	−1.728	−0.4585 to 0.1129	.22
	Intervention	−0.0202	−0.1245 to 0.0840	.69
**HCAI rates**				
	Year	0.3356	−1.4040 to 2,0751	.49
	Setting	−0.7453	−3.0057 to 1.5152	.29
	Design	−1.7453	−5.1746 to 8.6652	.39
	Intervention	3.8675	−9.3284 to 17.0634	.33

^a^HHC: hand hygiene compliance.

^b^HCAI: health care–associated infection.

**Table 3:** Sensitivity analysis for the outcomes.

Outcomes and subgroup		Study number	OR^a^ (95% CI)	*P* value	Quality of evidence
**HHC^b^**					
	All combined	29	1.56 (1.47-1.66)	<.001	⊕〇〇〇/very low
	Remove HH^c^ number <1000 (during intervention)	22	1.55 (1.45-1.65)	<.001	
**HCAI^d^ rates**					
	All combined	7	0.25 (0.19-0.33)	<.001	⊕⊕〇〇/low
	Remove Guo [28]	6	0.25 (0.18-0.34)	<.001	
	Remove Knudsen [32]	6	0.26 (0.19-0.34)	<.001	
	Remove Liu [37]	6	0.26 (0.20-0.34)	<.001	
	Remove McCalla [40]	6	0.28 (0.18-0.42）	<.001	
	Remove Wei [48]	6	0.25 (0.19-0.33)	<.001	
	Remove Xu [13]	6	0.22 (0.17-0.30)	<.001	
	Remove Zhu [49]	6	0.25 (0.18-0.33)	<.001	
**Detection rate of MDRO^e^**					
	All combined	6	0.53 (0.27-1.04)	.07	⊕⊕〇〇/low
	Remove Kato [14]	5	0.65 (0.31-1.32)	.23	
	Remove Liu [36]	5	0.71 (0.47-1.07)	.10	
	Remove Marra [38]	5	0.47 (0.23-0.97)	.04	
	Remove Shao [45]	5	0.49 (0.16-1.46)	.20	
	Remove Sun [46]	5	0.54 (0.26-1.10)	.09	
	Remove Xu [13]	5	0.45 (0.21-0.97)	.04	

^a^OR: odds ratio.

^b^HHC: hand hygiene compliance.

^c^HH: hand hygiene.

^d^HCAI: health care–associated infection.

^e^MDRO: multidrug-resistant organisms.

#### HCAI Rates

**Figure 3:**
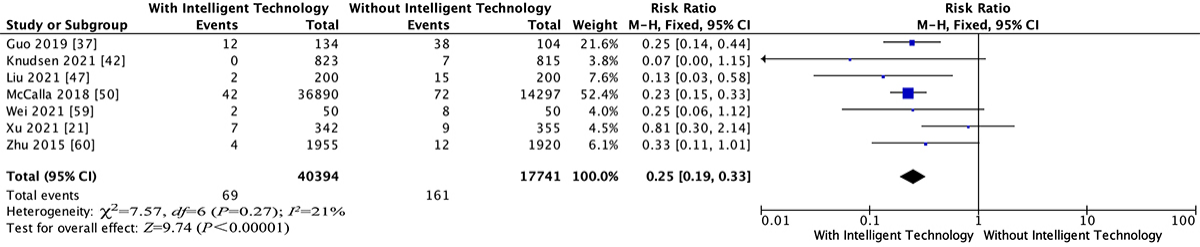
Forest plots for the outcome of health care–associated infection rates.

Seven trials reported this outcome. A fixed-effects model was used because of the low heterogeneity (*I^2^*=21%, *P*=.27; Figure 3) [21,37,42,47,50,59,60]. The pooled results showed that the intelligent technology interventions had a beneficial effect on HCAI rates (RR 0.25, 95% CI 0.19-0.33, *P*<.001; Figure 3). The sensitivity analysis obtained by removing one article at a time did not materially change these results (Table 3). The results of the Egger test showed no publication bias (*t*=0.11, *P*=.92>.05; Figure S3 in Multimedia Appendix 2). Using the GRADE summary of evidence, the quality of evidence was low and downgraded for high risk of bias and imprecision.

#### Detection Rate of MDRO

**Figure 4:**
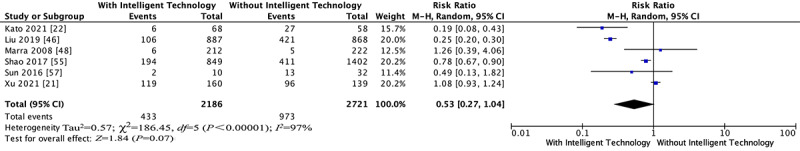
Forest plots for the outcome of multidrug-resistant organism detection rates.

Six studies examined the effects of intelligent technology interventions for HH on the detection rate of MDRO. A random-effects model was performed because of the significant heterogeneity for this outcome (*I^2^*=97%, *P*<.001; Figure 4) [21,22,46,48,55,57]. As shown in the forest plot, the RR exhibited a combined effect of 0.53 (95% CI 0.27-1.04, *P*=.07; Figure 4). The results of the meta-regression analysis indicated that the prespecified covariates had no effects on the detection rate of MDRO (Table 2). However, sensitivity analysis performed by removing one article at a time showed opposite results after 2 articles were removed (Table 3). The results of the Egger test showed no publication bias (*t*=−0.50, *P*=.64>.05; Figure S4 in Multimedia Appendix 2). Using the GRADE summary of evidence, the quality of evidence was very low and downgraded for high risk of bias and imprecision.

## Discussion

### Principal Findings

This systematic review appraised evidence from 36 studies evaluating the effects of intelligent technology interventions for HH on the behavior of HCWs, nosocomial infection rates, and MDRO detection rates. All studies, except 2 [48,58], were conducted after 2013, indicating a growing interest in applying intelligent technology for the management of HH. Our synthesized findings from the meta-analysis suggested that intelligent technology interventions for HH had a positive effect on HHC and contributed to the decrease in HCAI rates. This may be because most of the intelligent technology interventions provided reminders and real-time feedback to improve the HH awareness and habits of HCWs [50,61], further reducing infections [63]. However, our study could not determine the sustainability of the impact of intelligent technology interventions on HHC. Studies have shown that after the abolition of intelligent technology interventions, HHC dropped significantly, and the intelligent interaction between equipment and HCWs and direct personal feedback were important methods for improving the sustainability of HHC [64,65]. According to the analysis of the characteristics of the included literature, there were various methods, including instant prompts and feedback. Nevertheless, each type of reminder was associated with specific drawbacks, such as audible reminders that could interrupt a patient’s rest [66,67]. It was also challenging to effectively provide feedback to help HCWs understand the situation according to their needs and different educational backgrounds [68,69].

In contrast, our research focused on the outcomes of intelligent technology interventions for HH and showed no effect on MDRO detection rates. However, these results must be interpreted with caution because of the statistical heterogeneity (>90%), heterogeneity in terms of publication year, study design and interventions delivered (type of components), and unstable sensitivity analysis. At present, there is still controversy about the relationship between the improvement of HH behaviors and the detection rate of MDRO [70]. Studies found that the change in the MDRO detection rate was related to the length of time to improve HHC [71,72]. Improving HHC in a short period of time had no effect on the MDRO detection rate, and there was a delay effect. Studies have pointed out that this may be due to the nonlinear relationship between HHC and MDRO prevalence [72].

### Comparison With Other Studies

We were aware of 4 reviews of intelligent technology interventions for HH on the outcomes of HHC and HCAI rates. Previous systematic reviews led to different and incomplete conclusions. Two studies evaluated published articles indicating that technology systems could significantly improve HHC among health care professionals [65,73], in agreement with our results. However, the review by Srigley et al [61] indicated that 1 study evaluating the impact of electronic and video monitoring systems with a minimal potential for bias presented the smallest effect for HHC. We found only 1 systematic review showing that electronic and video monitoring systems have the potential to prevent HCAIs, but the results are not supported by sufficient data [74].

In addition to the 3 outcomes of this study, we noticed that some studies focused on the barriers to the application of intelligent technology for HH. Two systematic reviews found that usage anxiety, privacy, and confidentiality were key elements influencing the acceptance of intelligent technology interventions by HCWs [65,75]. They were concerned about potential risks posed by intelligent technology such as wearable sensors that could cause hand contamination and radio-frequency interference [76,77]. Some of HCWs perceived that these intelligent technology interventions using video cameras to monitor all 5 moments of HH would invade their and the patient’s privacy [11]. There were studies that suggested that a camera could be placed on the chest of HCWs that was aimed at their hands rather than installing cameras in the environment [78,79]. However, none of the included studies mentioned privacy protection before implementing intelligent technology interventions for HH. Another systematic review, which discussed costs, found that implementing intelligent technology interventions for HH in health care facilities would entail high costs, including equipment installation and maintenance costs, and that it was not realistic to install the camera system in community settings [4].

### Quality of Evidence

We assessed the quality of evidence for this study based on the GRADE classification. The quality of the evidence for the outcomes of the 3 studies was low, most of which were downgraded because of high risk of bias and imprecision. Therefore, the quality of evidence in this meta-analysis was low, and the results of the present meta-analysis were not strongly recommended.

### Strengths and Limitations of the Study

Several limitations should be noted in this meta-analysis. First, most of the included studies were quasi-experimental studies. Although only 2 of the studies conducted an RCT, blinding was difficult to implement because of the nature of intelligent technology interventions, which may potentially result in performance bias. Second, high heterogeneity was identified among studies in terms of results for the HHC and MDRO detection rates. Part of the heterogeneity may be due to differentiation in terms of populations and inconsistent inclusion and exclusion criteria among studies. Other sources of heterogeneity may be due to the diversity of intelligent technology interventions for HH and the lack of standardization, and system-related standards based on hardware limitations and WHO recommendations need to be established. Third, the sensitivity analysis showed unstable pooled results of MDRO detection rates. Further studies are needed to examine the effect of intelligent technology interventions for HH on this outcome. Fourth, our study performed no cost-benefit analysis of HH, and the results were unclear with respect to the rate of correct HH steps and the long-term sustainability of intelligent technology interventions. These limitations should be considered in future research.

Nevertheless, this meta-analysis strictly followed the PRISMA statement and applied a rigorous search strategy to identify potential studies in all available databases to ensure the generalizability of the results. Moreover, we included a relevantly large number of studies and sample sizes from various geographic areas, substantially enhancing the internal and external validity of the meta-analysis. This is the first meta-analysis to evaluate the impact of intelligent technology interventions for HH on multiple outcomes, which could provide valuable evidence to encourage intelligent technology application to improve clinical and nursing outcomes.

### Conclusions

Improving HH behaviors is an important part of hospital management, and it is of great significance to patients and HCWs. The application of intelligent technology to HH involves the innovation of management methods. This systematic review determined that intelligent technology interventions for HH had an important role in improving HHC and reducing HCAI rates, but it could not be determined whether it had an effect on the MDRO detection rate. However, low-quality evidence and important heterogeneity were observed. Important directions for future work are to further verify the 3 outcomes through high-quality research and conduct more research to evaluate the impacts of intelligent technology interventions on the long-term sustainability, cost-effectiveness, and rate of correct HH.

## Appendix

Multimedia Appendix 1 PRISMA 2020 checklist.

Multimedia Appendix 2 Supplementary material.

## Abbreviations

GRADE: Grading of Recommendations, Assessment, Development, and Evaluation
HCAI: health care–associated infection
HCW: health care worker
HH: hand hygiene
HHC: hand hygiene compliance
MDRO: multidrug-resistant organism
PRISMA: Preferred Reporting Items for Systematic Reviews and Meta-Analyses
RCT: randomized controlled trial
ROBINS-I: Risk Of Bias In Non-randomised Studies—of Interventions
RR: risk ratio
WHO: World Health Organization

## Data Availability

All data generated or analyzed during this study are included in this published article.
