# Supplementary material for: Effects of the Implementation of Intelligent Technology for Hand Hygiene in Hospitals: Systematic Review and Meta-analysis
Source: J Med Internet Res. 2023 May 29;25:e37249. doi: 10.2196/37249 (PMC10262028; doi:10.2196/37249)

**Supplementary Material**

**Effects of Hand Hygiene Intelligent Technology Implementation in Hospitals: A**

**Systematic Review and Meta-analysis**

**CONTENTS**

**1. Textbox S1. Search strategies of databases.**

**2. Table S1. Data Extraction Form.**

**4. Table S2. Characteristics of the studies included in the meta-analysis.**

**5. Table S3. Risk of bias assessment of the included studies based on the ROBINS-I tool.**

**6. Figure S1. Risk of bias graph for the included studies based on the Cochrane Risk of Bias methodology.**

**7. Figure S2. Funnel plot for publication bias assessment of the included studies involving the outcome of hand hygiene compliance.**

**8. Figure S3. Funnel plot for publication bias assessment of the included studies involving the outcome of health care-associated infection rates.**

**9. Figure S4. Funnel plot for publication bias assessment of the included studies involving the outcome of multidrug-resistant organism detection rates.**

**Search strategies of databases**

*Medline:*

Search: ((("Hand Hygiene"[Mesh]) OR ("Hand Disinfection"[Mesh])) OR (((((((("hand hygiene"[Title/Abstract]) OR ("hand disinfection"[Title/Abstract])) OR ("hand sanitization"[Title/Abstract])) OR ("hand wash*"[Title/Abstract])) OR ("handwashing"[Title/Abstract])) OR ("hand clean*"[Title/Abstract])) OR ("hand scrubbing"[Title/Abstract])) OR ("surgical scrubbing"[Title/Abstract]))) AND ((((("Information Technology"[Mesh]) OR "Robotics"[Mesh]) OR "Artificial Intelligence"[Mesh]) OR "Neural Networks, Computer"[Mesh]) OR ((((((((techni*[Title/Abstract]) OR (devic*[Title/Abstract])) OR (equipment*[Title/Abstract])) OR (system*[Title/Abstract])) OR (electric[Title/Abstract])) OR (robotics[Title/Abstract])) OR ("artificial intelligence"[Title/Abstract])) OR (technology[Title/Abstract])))

*EMBASE:*

#1 'hand washing'/exp OR 'hand washing'

#2 'hand disinfection'/exp OR 'hand disinfection'

#3 'technology'/exp OR 'technology'

#4 'artificial intelligence'/exp OR 'artificial intelligence'

#5 'robotics'/exp OR 'robotics'

#6 'electrical equipment'/exp

#7 'hand hygiene':ab,ti OR 'hand disinfection':ab,ti OR 'hand sanitization':ab,ti OR 'hand wash*':ab,ti OR handwashing:ab,ti OR 'hand clean*':ab,ti OR 'hand scrubbing':ab,ti OR 'surgical scrubbing':ab,ti

#8 techni*:ab,ti OR devic*:ab,ti OR equipment*:ab,ti OR system*:ab,ti OR electric:ab,ti OR robotics:ab,ti OR 'artificial intelligence':ab,ti OR technology:ab,ti

#9 #1 OR #2 OR #7

#10 #3 OR #4 OR #5 OR #6 OR #8

#11 #9 AND #10

*CINAHL:*

S1 (MH "Handwashing+") 
S2 (MH "Surgical Scrubbing")

S3 (MH "Digital Technology+") OR (MH "Information Technology") OR (MH "Technology")

S4 (MH "Robotics+")

S5 (MH "Artificial Intelligence")

S6 (MH "Electrical Equipment and Supplies")

S7 TI "hand hygiene" OR AB "hand hygiene"

S8 TI "hand disinfection" OR AB "hand disinfection"

S9 TI "hand sanitization" OR AB "hand sanitization"

S10 TI "hand wash*" OR AB "hand wash*"

S11 TI "hand clean*" OR AB "hand clean*"

S12 TI "hand scrubbing" OR AB "hand scrubbing"

S13 TI "surgical scrubbing" OR AB "surgical scrubbing"

S14 TI handwashing OR AB handwashing

S15 TI techni* OR AB techni*

S16 TI devic* OR AB devic*

S17 TI equipment* OR AB equipment*

S18 TI system* OR AB system*

S19 TI electric OR AB electric

S20 TI robotics OR AB robotics

S21 TI technology OR AB technology

S22 TI "artificial intelligence" OR AB "artificial intelligence"

S23 S1 OR S2 OR S7 OR S8 OR S9 OR S10 OR S11 OR S12 OR S13 OR S14

S24 S3 OR S4 OR S5 OR S6 OR S15 OR S16 OR S17 OR S18 OR S19 OR S20 OR S21 OR S22

S25 S23 AND S24

*CENTRAL and CDSR:*

#1 MeSH descriptor: [Hand Hygiene] this term only

#2 MeSH descriptor: [Hand Disinfection] this term only

#3 ("hand hygiene" OR "hand disinfection" OR "hand sanitization" OR "hand wash*" OR handwashing OR "hand clean*" OR "hand scrubbing" OR "surgical scrubbing"):ti,ab,kw

#4 MeSH descriptor: [Information Technology] this term only

#5 MeSH descriptor: [Robotics] this term only

#6 MeSH descriptor: [Artificial Intelligence] this term only

#7 MeSH descriptor: [Neural Networks, Computer] this term only

#8 MeSH descriptor: [Technology] this term only

#9 (techni* OR devic* OR equipment* OR system* OR electric OR robotics OR "artificial intelligence" OR technology):ti,ab,kw

#10 #1 ORr #2 OR #3

#11 #4 OR #5 OR #6 OR #7 OR #8 OR #9

#12 #10 AND #11

*Web of Science Core Collection:*

#1 TI="hand hygiene" OR AB="hand hygiene"

#2 TI="hand disinfection" OR AB="hand disinfection"

#3 TI="hand sanitization" OR AB="hand sanitization"

#4 TI="hand wash*" OR AB="hand wash*"

#5 TI=handwashing OR AB=handwashing
#6 TI="hand clean*"OR AB="hand clean*"
#7 TI="hand scrubbing" OR AB="hand scrubbing"

#8 TI="surgical scrubbing" OR AB="surgical scrubbing"

#9 #1 OR #2 OR #3 OR #4 OR #5 OR #6 OR #7 OR #8
#10 TI=techni* OR AB=techni*

#11 TI=devic* OR AB=devic*

#12 TI=equipment* OR AB=equipment*

#13 TI=system* OR AB=system*
#14 TI=electric OR AB=electric

#15 TI=robotics OR AB=robotics

#16 TI="artificial intelligence" OR AB="artificial intelligence"

#17 TI=technology OR AB=technology

#18 #10 OR #11 OR #12 OR #13 OR #14 OR #15 OR #16 OR #17

#19 #9 AND #18

*Chinese Academic Journal:*

#1 [(篇关摘=手卫生) OR (篇关摘=洗手) OR (篇关摘=手消毒)](https://kns.cnki.net/kns8/AdvSearch?id=50&dbcode=CFLS&searchtype=gradeSearch&ishistory=1" \o "(篇关摘=手卫生) OR (篇关摘=洗手) OR (篇关摘=手消毒)" \t "/Users/zhanggaoxing/Documents\\x/_blank)

#2 [(篇关摘=技术) OR (篇关摘=系统) OR (篇关摘=智能) OR (篇关摘=工具) OR (篇关摘=装置) OR (篇关摘=设备) OR (篇关摘=仪器)](https://kns.cnki.net/kns8/AdvSearch?id=51&dbcode=CFLS&searchtype=gradeSearch&ishistory=1" \o "(篇关摘=技术) OR (篇关摘=系统) OR (篇关摘=智能) OR (篇关摘=工具) OR (篇关摘=装置) OR (篇关摘=设备) OR (篇关摘=仪器)" \t "/Users/zhanggaoxing/Documents\\x/_blank)

#3 #1AND #2

**Table S1. Study Selection and Data Extraction Form**

Study Selection and Data Extraction Form

Name of author extracting data: Date form completed:

Party1: General study information

| Items | Data | Notes |
| --- | --- | --- |
| Title |  |  |
| Study ID |  |  |
| Authors |  |  |
| Journal |  |  |
| Year |  |  |
| Country |  |  |
| COI |  |  |

Party 2: Study Selection

| Items | Choose | Notes |
| --- | --- | --- |
| Type of studies:  Can this study be considered a randomised controlled trial or quasi-experimental trail ? | Yes □  Unclear □  No □ |  |
| Types of participants:  Were the participants HCWs or the included patients adults (18 years old or older) ? | Yes □  Unclear □  No □ |  |
| Interventions:  Did study evaluate the effectiveness of IT-related intervention alone or in combination with usual care compared to placebo or usual methods? | Yes □  Unclear □  No □ |  |
| Outcomes:  Did the study report at least one clinical endpoint such as HHC、HCAI rates or MDRO detection rates ? | Yes □  Unclear □  No □ |  |
| Conclusion:  Do not proceed if any of the previous answers are ‘No’.  □Included  □Excluded and listed in excluded table  □More information needed before inclusion decision (specify) | | |

Party 3: Study details

| Inclusion/Exclusion Criteria: | | | |  |
| --- | --- | --- | --- | --- |
|  | RCT or Non-randomized controlled trial | | One-group pretest–posttest controlled trial |  |
|  | Intervention | Control |  |  |
| Number of Participants |  |  |  |  |
| Type of Participants |  |  |  |  |
| Age, mean (SD)  Median (IQR) |  |  |  |  |
| Male n (%)  Female n (%) |  |  |  |  |
| Setting |  | | | |
| IT Intervention (component? How long?) | | | | |
| Control | | | | |
| Follow up | | | | |
| outcomes | | | | |

**Abbreviation:** HHC, hand hygiene compliance; HCAI, health care-associated infection; MDRO, multi-Drug resistant organisms; HCWs, health care workers; IT, Intelligent Technology.

**Table S2.** **Characteristics of the studies included in the meta-analysis.**

| **Author/Year** | **Country** | **Study design** | **Setting** | **Participants(N)** | **Summary of IT intervention** | **Data collection period** | **Study outcomes** |
| --- | --- | --- | --- | --- | --- | --- | --- |
| Al Salman 2015 [62] | Canada | One-group pretest–posttest quasi-experimental design | CCU | All HCWs  (N= NI)  Age: NI | Electronic monitoring system (2, 3, 4)  • Electronic HH counting device (hand sanitizer dispenser)  • Real-time feedback  • A wearable badge | • Pre-intervention  • 28 days | ① HHC  • WHO moment 1 and 4 |
| Arai 2016 [30] | Japan | One-group pretest–posttest quasi-experimental design | 28 clinical departments | N=280 physicians, Age: NI | • Electronic HH counting device (hand sanitizer dispenser)  • Remote video auditing  (2) | • 1 months bofore intervention  • 1 month, 2 months | ① HHC  • Outpatient visit records |
| Armellino 2013 [31] | USA | One-group pretest–posttest quasi-experimental design | SICU | All HCWs  (N= NI, Age: NI) | Remote video auditing (2, 4)  • Remote video auditing  • Real-time feedback | • Pre-intervention  • 4 months, 12 months | ① HHC  • Patient room entry/exit events |
| Bai 2021 [32] | China | Non-randomized controlled trial | Hemodialysis Center | N= 2 doctors, 19 nurses  Age: 25.32±2.26 years | Internet management system (1, 2, 3, 4)  • A wearable badge  • Electronic HH counting device  • Performance reminders  • Data analysis | Intervention/control group: 5months | ① HHC  • WHO 5 moments  ② Consumption of hand sanitizer |
| Boyce, Cooper 2019 [33] | USA | One-group pretest–posttest quasi-experimental design | SICU and a general medical ward | N=126 HCWs, Age: NI | Automated HH monitoring systems (1, 2, 3, 4)  • Electronic HH counting device (hand sanitizer dispenser)  • A wearable badge  • Performance reminders  • Data analysis | • 7 months before the intervention  • 3 months and 1 week | ① HHC  • Patient room entry/exit events |
| Boyce, Laughman 2019 [34] | USA | One-group pretest–posttest quasi-experimental design | 2 medical-surgical wards, 1 step-down ward, and 1 medical-surgical ICU | All HCWs  (N= NI)  Age: NI | Automated HH monitoring systems (AHHMSs) (1, 2, 3, 4)  • A wearable badge  • Electronic HH counting device (hand sanitizer dispenser)  • Performance reminders  • Record analysis | • 1 month before installing AHHMS  • 12 months (AHHMS with administrative strategy 1)  • 5 months (AHHMS with administrative strategy 1 and visits)  • 14 months (AHHMS with administrative strategies 1 and 2 plus visits) | ① HHC  • Patient room entry/exit events |
| Brotfain 2017 [35] | Israel | Non-randomized controlled trial | ICU | N=36 HCWs, Age: NI | HH monitoring programs (2)  • Online\real-time television camera monitoring | Intervention/control group:  • 28 months | ① HHC  • WHO 5 moments |
| Generoso 2022 [36] | Brazil | Non-randomized controlled trial | ICU | All HCWs  (N= NI)  Age: NI | An invasive device sensor (1, 2, 3, 4)  • A wearable radio badge  • Electronic HH counting device (hand sanitizer dispenser)  • Performance reminders  • Record analysis | Intervention/control group:  • 6 months | ① HHC  • WHO 5 moments |
| Guo 2019 [37] | China | One-group pretest–posttest quasi-experimental design | ICU | N=38 HCWs, Age: NI | Smart HH management system (1, 2, 3, 4)  • A wearable badge  • Electronic HH counting device (hand sanitizer dispenser)  • Record analysis | • 1 year bofore intervention  • 4 months | ① HHC  • Patient room entry/exit events  ② HCAI rates |
| Higgins 2013 [38] | Ireland | One-group time series quasi-experimental design | All unites in one hosipal | All HCWs  (N= NI)  Age: NI | SureWash (2, 5)  •E-learning HH game  •ATP monitoring of HH techniques | • Pre-intervention,3 months, 6 months, 9 months, 12 months (general HH intervention)  • 3 months, 6 months, 9 months,12 months(SureWash intervention) | ① HHC  • Patient room entry/exit events |
| Hu 2019 [39] | China | One-group pretest–posttest quasi-experimental design | ICU | N=106 HCWs  Age: NI | Smart HH Management (1, 2, 3, 4)  •A wearable badge  •Electronic HH counting device (hand sanitizer dispenser)  •Performance reminders  •Record analysis | • 1 month bofore intervention  • 1 month | ① HHC  • Patient room entry/exit events |
| Kato 2021 [22] | Japan | Non-randomized controlled trial | Outpatient room | N=134 physicians  Age: NI | Automated monitoring system (2, 3, 4)  • Wireless ﬁdelity (WIFI)  • Data collection and analysis  • Real-time feedback | Intervention/control group: 44 days | ① MDRO detection rates |
| Kerbaj 2017 [40] | France | One-group pretest–posttest quasi-experimental design | Infectious diseases department | N=18 HCWs  Age: NI | MediHandTrace (1, 2, 3)  • Personal electronic microchip-tagged shoes  • Electronic HH counting device (hand sanitizer dispenser)  • Performance reminders  • Record analysis | • 360 days bofore intervention  • 221 days | ① HHC  • Patient room entry/exit events |
| Khan 2017 [41] | Pakistan | One-group pretest–posttest quasi-experimental design | Operating room | All HCWs  (N= NI)  Age: NI | A remote video auditing system (2, 3, 4)  • Real-time feedback  • Remote video auditing  • Record analysis | • 4 months bofore intervention  • 4 months, 8 months, 12 months | ① HHC  • Patient room entry/exit events |
| Knudsen 2021 [42] | Denmark | One-group pretest–posttest quasi-experimental design | Nephrology department | N=77 nurses, 22 doctors | An electronic hand hygiene (1, 2, 3)  • A wearable badge  • Electronic HH counting device (hand sanitizer dispenser)  • Record analysis | • 4months bofore intervention  • 4 months, 3 months | ① HCAI rates |
| Kwok 2015 [43] | Australia | One-group pretest–posttest quasi-experimental design | 18 inpatient medical units (surgery, obstetrics, emergency department) | N=789 HCWs  Age: NI | Automated training technology (2,5)  • 7 steps for HH  • Automated record compliance | • 1year Pre-intervention  •1 year During the intervention  • 1 year After the intervention | ① HHC  • Patient room entry/exit events |
| Lacey 2020 [44] | UK | One-group pretest–posttest quasi-experimental design | Surgical ward | All HCWs  (N= NI)  Age: NI | • Automated video auditing  • Real-time feedback  (2,4) | • 10 days before the intervention  • 32 days, 64 days, 6 days | ① HHC  • Patient room entry/exit events  ② HH correct rates |
| Leis 2020 [45] | Canada | One-group pretest–posttest quasi-experimental design | 26 in-patient units (14 medicine, 10 surgery, 1 mixed, 1 chronic care) | All HCWs  (N= NI)  Age: NI | Group electronic monitoring (1,4)  • Visual reminders  • Email feedback | • 3 months bofore intervention  • 1month, 10 months | ① HHC  • Patient room entry/exit events |
| Leis 2022 [15] | Canada | One-group pretest–posttest quasi-experimental design | ICU | All HCWs  (N= NI)  Age: NI | Electronic hand hygiene monitoring systems (1, 2, 3)  • A wearable badge  • Electronic HH counting device (hand sanitizer dispenser)  • Record analysis | • 60 days bofore intervention  • 55 days | ① HHC  • WHO 5 moments |
| Liu 2019 [46] | China | Non-randomized controlled trial | ICU | N=68 HCWs Age: (Intervention/Control) 29.22±1.94years/29.14±1.46years | Hand hygiene management system (1, 2, 3)  • A wearable device  • Electronic HH counting device (hand sanitizer dispenser)  • Real-time recording | • 1 year bofore intervention  • 1 year | ① MDRO detection rates  ② Consumption of hand sanitizer |
| Liu 2021 [47] | China | One-group pretest–posttest quasi-experimental design | Multiple clinical departments | N=128 HCWs ,400 patients  Age: 32.5±3.2years, 32.25±3.78years | Intelligent Hand Hygiene Management System (1, 2, 3)  • A wearable badge  • Electronic HH counting device (hand sanitizer dispenser)  • Record analysis | • 12 months bofore intervention  • 12 months | ① HHC  • WHO 5 moments  ② HCAI rates  ③ Satisfaction rates of patients  ④ Awareness rates |
| Marra 2008 [48] | USA | Non-randomized controlled trial | Adult step-down units | N= HCWs, 227 Patients  Age: NI | • Electronic HH counting device (hand sanitizer dispenser)  • Performance reminders  • Data analysis  (1, 2, 3) | NI | ① MDRO detection rates |
| Marra 2014 [49] | Brazil | One-group pretest–posttest quasi-experimental design | Two medical-surgical step-down wards | N= 20 HCWs  Age: NI | Wireless monitoring system (1, 2, 3, 4)  • A wearable badge  • Electronic HH counting device (hand sanitizer dispenser)  • Data analysis  • Real-time feedback | • 1 month before the intervention  • 8 months (including 1-month washout period) | ① HHC  • Patient area entry/exit events  ② Consumption of hand sanitizer |
| McCalla 2018 [50] | USA | non-randomized pre-post intervention study | All unites | N= 51187 patients  Mean age: 67 years | An automated HHC system (1, 2, 3)  • A wearable badge  • Electronic HH counting device (hand sanitizer dispenser)  • Record analysis | • 14 months bofore intervention  • 31 months | ① HCAI rates |
| McMullen 2022 [19] | USA | One-group pretest–posttest quasi-experimental design | All unites in 12 hospitals | All HCWs | Electronic monitoring system (1, 2, 3, 5)  • A wearable badge  • Electronic HH counting device (hand sanitizer dispenser)  • Performance reminders  • Record analysis  • Education | Intervention 3 years | ① HHC  • Restroom entry/exit events |
| Møller-Sørensen 2016 [51] | Denmark | One-group pretest–posttest quasi-experimental design | Four toilets in hospital | All HCWs | Hand-hygiene dispensing technology (2)  • Intelligent soap dispenser  • Hidden recorder | • 3 months bofore intervention  • 3months | ① HHC  • Restroom entry/exit events |
| Pan 2020 [52] | China | One-group pretest–posttest quasi-experimental design | ICU | N= 25 nurses, Mean age: 23-43 years | Intelligent Control Hand Hygiene IoT System (1, 2, 3)  • A wearable device  • Electronic HH counting device (hand sanitizer dispenser)  • Record analysis | • 1year bofore intervention  • 1 year | ① HHC  • Patient area entry/exit events |
| Pires 2021 [53] | Swiss | RCT | 12 wards (5 internal medicine, 5 geriatrics, 1 emergency department, 1 ambulatory unit) | N=97 HCWs (26 group 1, 22 group 2, 25 group 3, 24 group 4)  Age: 41.6±1.2year (group1), 44.6±9.2year (group2), 40.2±12.6year (group3), 41.9±12.2year (group4) | SmartRub (1, 2, 3)  • A wearable device  • Electronic HH counting device (hand sanitizer dispenser)  • Record analysis | • 1 month baseline  • 1 month transition periods before intervention  • 4 months intervention  • 1months follow-up period | ① HHC  • Patient area entry/exit events |
| Pong 2018 [54] | Canada | RCT | Rehabilitation unite | N=511 HCWs  Age: NI | Electronic monitoring system (1, 2, 3)  • A wearable badge  • Electronic HH counting device (hand sanitizer dispenser)  • Record analysis | Intervention/control group: 5 months | ① HHC  • Patient area entry/exit events |
| Shao 2017 [55] | China | Non-randomized controlled trial | ICU | N=63 nurses, 29 doctors  Age: 31.7±1.3 years | LINKWELL Hand hygiene (1, 2, 3) management system  • A wearable device  • Electronic HH counting device (hand sanitizer dispenser)  • Record analysis | Intervention/control group: 13 months | ① MDRO detection rates |
| Simonet 2022 [56] | Switzerland | One-group pretest–posttest quasi-experimental design | 46 unites | N=12460 nurses, 2833 doctors  Age: NI | “CleanHands” web application (3, 4)  • Real-time feedback  • Data collection and analysis | Intervention 7 months | ① HHC  • WHO 5 moments |
| Sun 2016 [57] | China | One-group pretest–posttest quasi-experimental design | ICU | N=29 HCWs  Age: NI | Intelligent alert and management system (1, 2, 3)  • A wearable badge  • Electronic HH counting device (hand sanitizer dispenser)  • Record analysis | • 6 months before intervention  • 6 months | ① HHC  • Patient area entry/exit events  ② MDRO detection rates  ③ HCAI rates |
| Venkatesh 2008 [58] | USA | One-group pretest–posttest quasi-experimental design | Hematology unit | All HCWs  (N= NI)  Age: NI | Automated devices (1, 2, 3)  • Electronic HH counting device (hand sanitizer dispenser)  • Performance reminders  • Record analysis | • 14 days bofore intervention  • 11days | ① HHC  • Patient area entry/exit events |
| Wei 2021 [59] | China | One-group pretest–posttest quasi-experimental design | ICU | N=50 HCWs  Age: NI | Hand hygiene intelligent monitoring system (1, 2, 3)  • A wearable badge  • Electronic HH counting device (hand sanitizer dispenser)  • Record analysis | • 1year bofore intervention  • 1 year | ① HHC  • Patient area entry/exit events  ② HCAI rates |
| Xu 2021 [21] | China | One-group pretest–posttest quasi-experimental design | Emergency ICU | N=54 HCWs, 697 patients  Age: NI | Internet of Things management system (1, 2, 3)  • A wearable badge  • Electronic HH counting device (hand sanitizer dispenser)  • record analysis | • 4 months before intervention  • 4 months | ① HHC  • Patient area entry/exit events  ② MDRO detection rates  ③ HCAI rates |
| Zhu 2015 [60] | China | One-group pretest–posttest quasi-experimental design | Operating room | N=254 HCWs  Age: NI | digital video monitoring (2, 3)  • Remote video auditing  • Record analysis | • 6 months before intervention  • 6 months | ① HCAI rates |

**Abbreviation:** CCU, coronary care unit; HCWs, health care workers; IT, Intelligent Technology; HH, hand hygiene; HHC, hand hygiene compliance; HCAI, health care-associated infection; MDRO, multi-drug resistant organism; SICU, surgery intensive care unit; NI, no information; WHO, World Health Organization.

1, perforemence reminders: Prompting healthcare workers either through a wearable devices、electronic communications or other methods to remind them about hand hygiene; 2, Electronic HH counting and remote monitoring: Devices was installed on the handwashing equipment to remotely monitor and capture HH data; 3, Data processing: Upload data to a database and analysis; 4, feedback: Provide compliance feedback to staff via mobile message、email or other methods; 5, Education: Providing educational programme on correct procedures of HH.

WHO moment 1: Before touching a patient; WHO moment 2: Before clean/aseptic procedure; WHO moment 3: After body fluid exposure risk; WHO moment 4: After touching a patient; WHO moment 5: After touching patient surroundings.

**Table S3. Risk of bias assessment of the included studies based on the ROBINS-I tool.**

| **Author** | **Bias due to confounding** | **Bias in the selection of participants into the study** | **Bias in the classification of interventions** | **Bias due to deviations from intended interventions** | **Bias due to missing data** | **Bias in outcome measurements** | **Bias in selection of the reported results** | **Overall Risk-of-bias judgement** |
| --- | --- | --- | --- | --- | --- | --- | --- | --- |
| Al Salman et al. [62] | moderate | low | low | moderate | low | moderate | moderate | moderate |
| Arai et al. [30] | moderate | moderate | serious | serious | low | moderate | moderate | serious |
| Armellino et al. [31] | moderate | moderate | moderate | moderate | low | moderate | moderate | moderate |
| Bai et al. [32] | low | moderate | low | moderate | low | moderate | moderate | moderate |
| Boyce et al. [33] | low | low | low | moderate | low | moderate | moderate | moderate |
| Boyce, Laughman et al. [34] | moderate | serious | low | low | low | moderate | moderate | serious |
| Brotfain et al. [35] | low | low | low | low | low | moderate | moderate | moderate |
| Generoso et al. [36] | moderate | low | low | low | low | moderate | moderate | moderate |
| Guo et al. [37] | moderate | moderate | low | Serious | low | moderate | moderate | moderate |
| Higgins et al. [38] | moderate | low | low | low | low | moderate | moderate | moderate |
| Hu et al. [39] | low | low | low | moderate | low | moderate | moderate | moderate |
| Kato et al. [22] | low | low | moderate | serious | low | moderate | moderate | serious |
| Kerbaj et al. [40] | low | low | Serious | low | low | moderate | moderate | serious |
| Khan et al. [41] | low | low | low | low | low | moderate | moderate | moderate |
| Knudsen et al. [42] | moderate | low | low | moderate | low | moderate | moderate | moderate |
| Kwok et al. [43] | moderate | low | low | moderate | low | moderate | moderate | moderate |
| Lacey et al. [44] | moderate | moderate | serious | moderate | low | moderate | moderate | serious |
| Leis et al. [45] | moderate | low | low | low | low | moderate | moderate | moderate |
| Leis et al. [15] | moderate | low | moderate | low | low | moderate | moderate | moderate |
| Liu et al. [46] | low | moderate | low | serious | low | moderate | moderate | serious |
| Liu et al. [47] | moderate | moderate | low | serious | low | moderate | low | serious |
| Marra et al. [48] | low | low | low | moderate | low | moderate | moderate | moderate |
| Marra 2014 [49] | low | low | low | moderate | low | moderate | moderate | moderate |
| McCalla et al. [50] | low | low | low | low | low | moderate | moderate | moderate |
| McMullen et al. [19] | serious | low | moderate | moderate | moderate | moderate | moderate | serious |
| Møller-Sørensen et al. [51] | moderate | low | low | moderate | low | moderate | moderate | moderate |
| Pan et al. [52] | low | low | moderate | moderate | low | moderate | moderate | moderate |
| Shao et al. [55] | low | moderate | low | moderate | low | moderate | low | moderate |
| Simonet et al. [56] | moderate | moderate | low | low | low | moderate | moderate | moderate |
| Sun et al. [57] | low | low | low | moderate | low | moderate | low | moderate |
| Venkatesh et al. [58] | low | low | low | moderate | low | moderate | moderate | moderate |
| Wei et al. [59] | moderate | low | low | moderate | low | moderate | moderate | moderate |
| Xu et al. [21] | low | low | low | serious | low | moderate | low | serious |
| Zhu et al. [60] | low | low | low | moderate | low | moderate | moderate | moderate |

**Figure S1. Risk of bias graph for the included studies based on the Cochrane Risk of Bias methodology.**


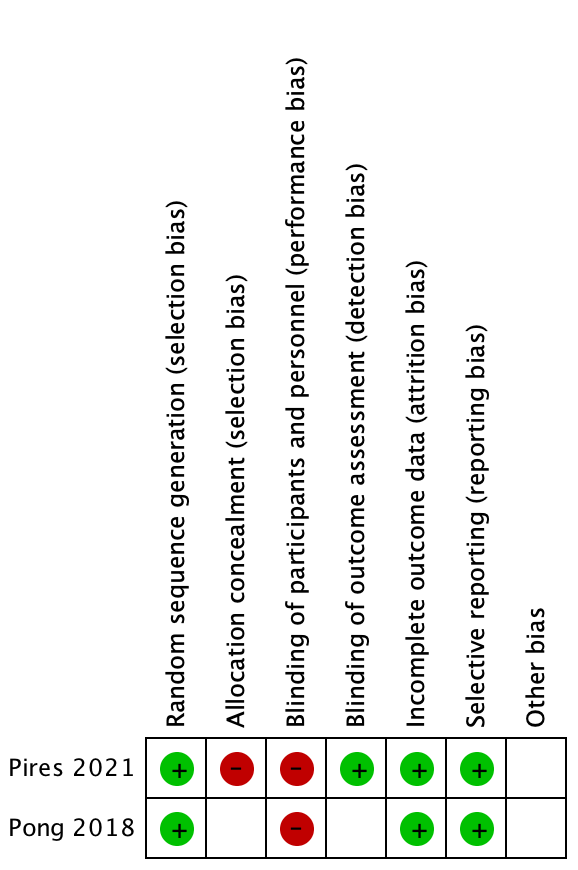


**Figure S2. Funnel plot for publication bias assessment of the included studies involving the outcome of hand hygiene compliance.**


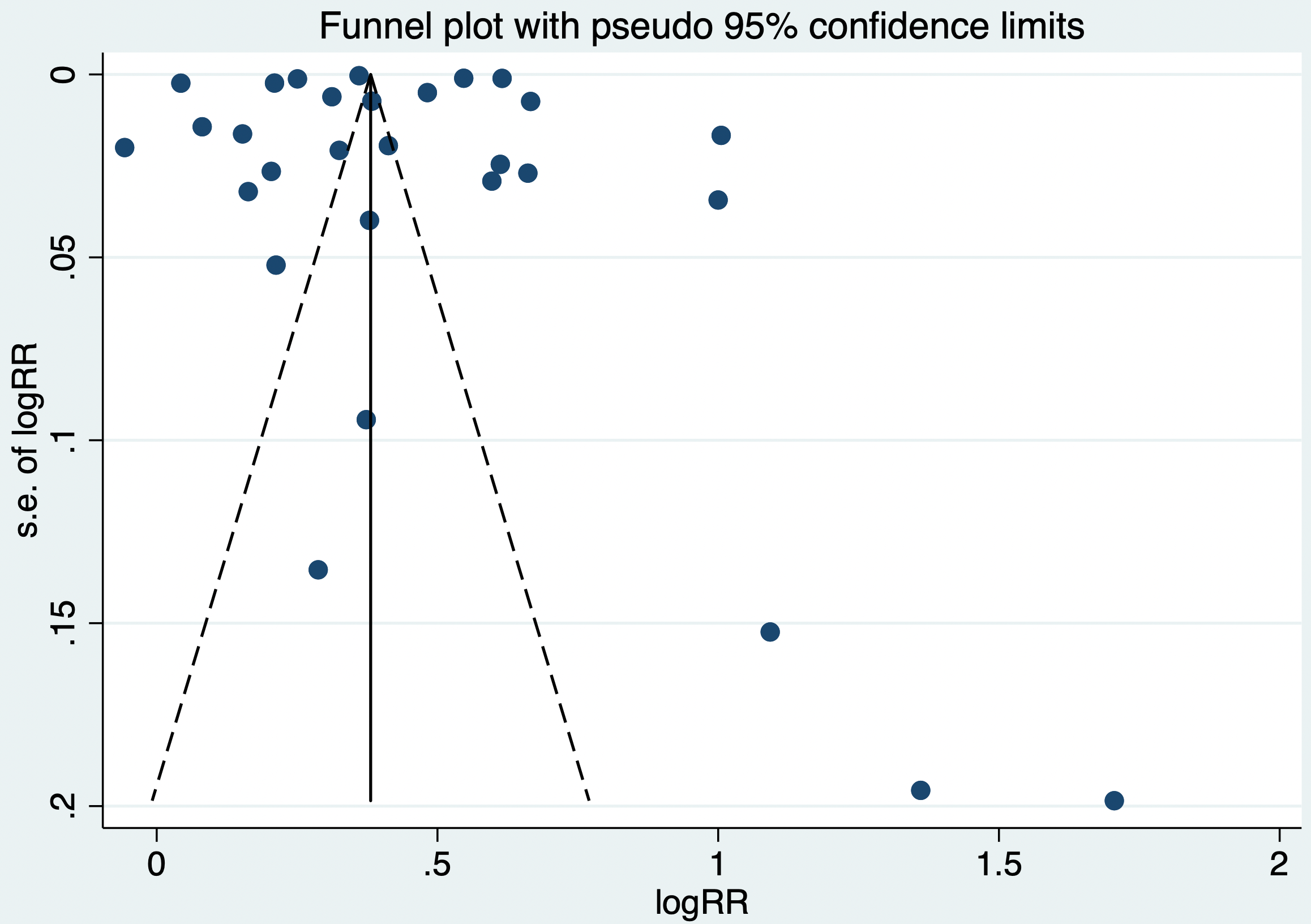


**Figure S3. Funnel plot for publication bias assessment of the included studies involving the outcome of health care-associated infection rates.**


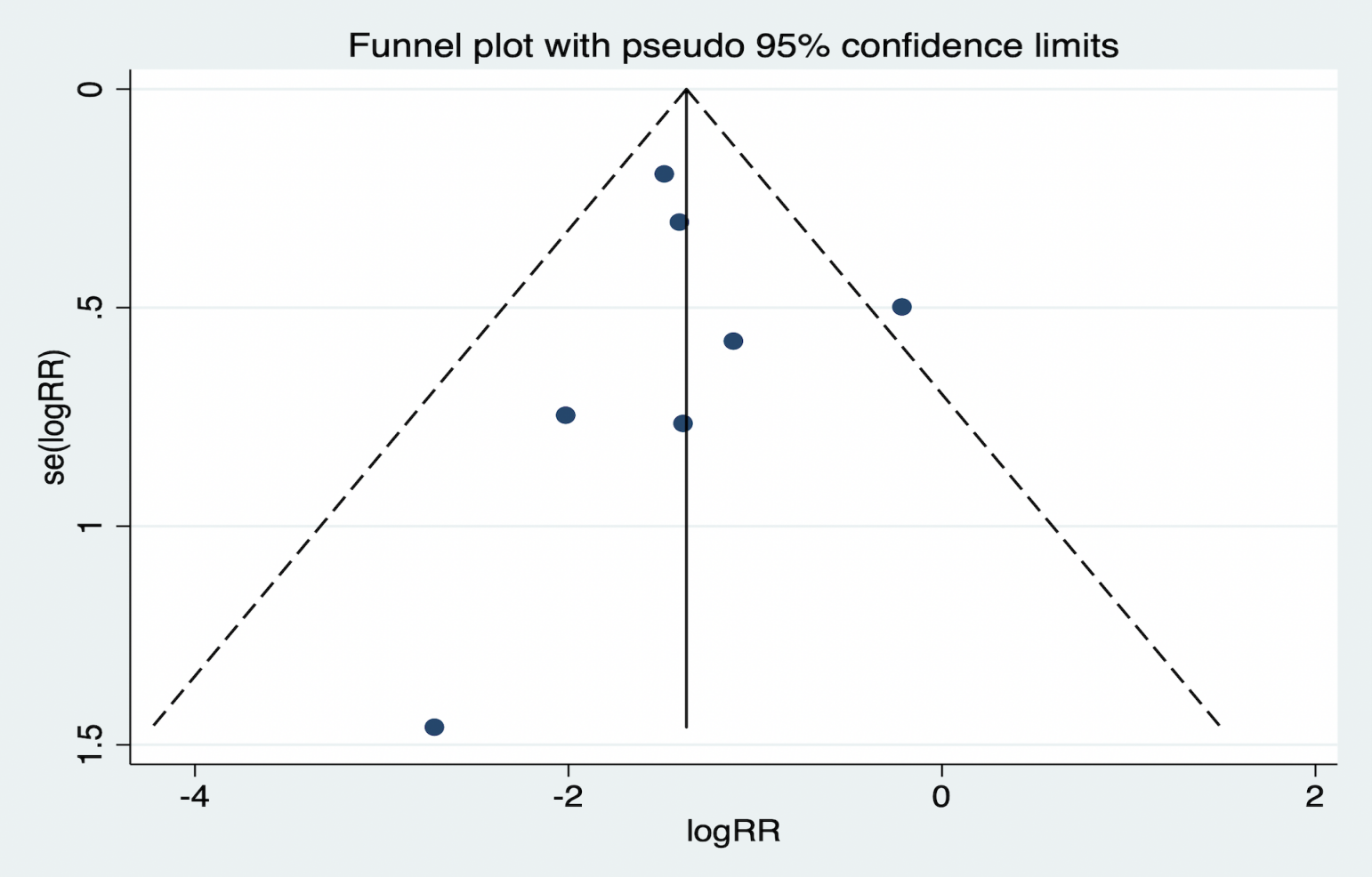


**Figure S4. Funnel plot for publication bias assessment of the included studies involving the outcome of multi-drug resistant organism detection rates.**


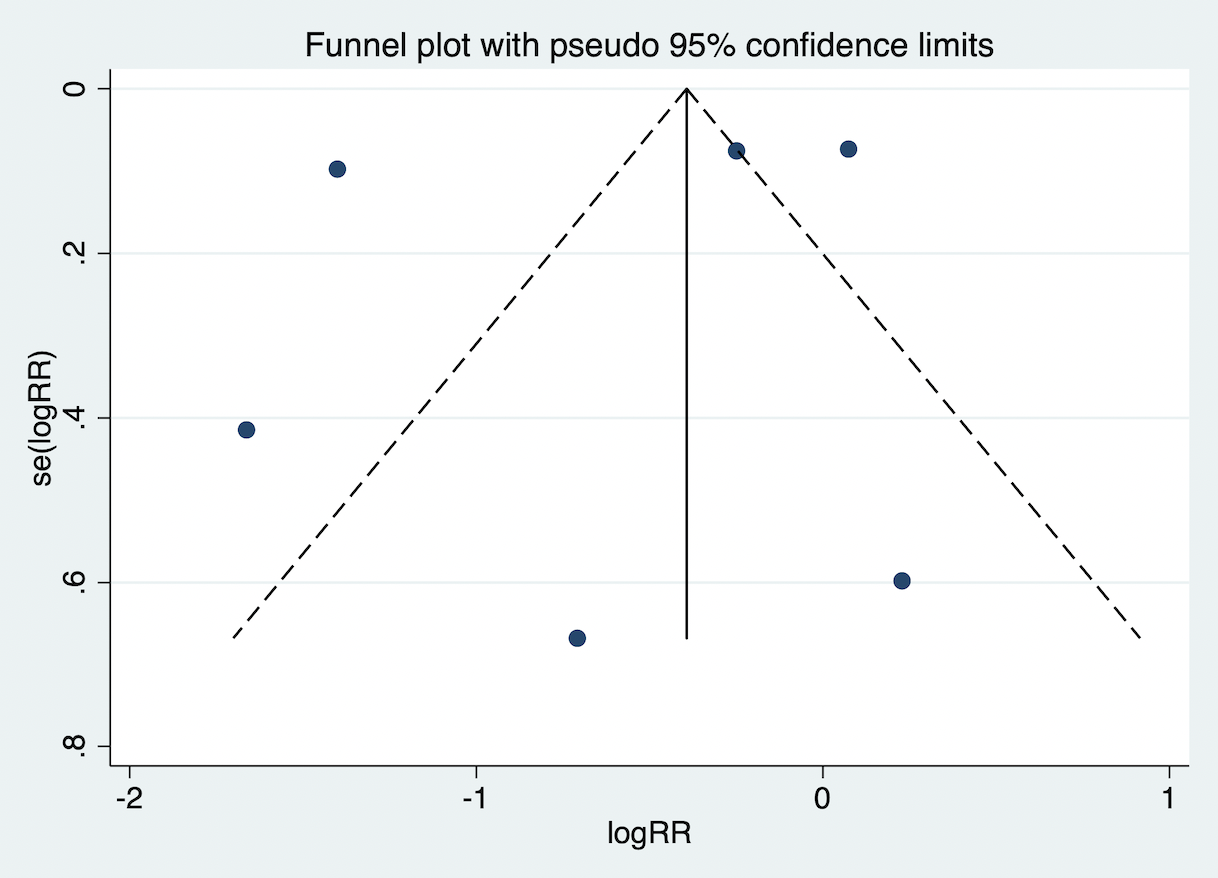

Supplement: Multimedia Appendix 2 [file jmir_v25i1e37249_app2.docx]
